# Impact of Radiofrequency Ablation and Antiarrhythmic Medications on the Quality of Life of Patients with Supraventricular Tachycardias: Preliminary Validation of the Greek Version of the Umea22 (U22) Questionnaire

**DOI:** 10.1155/2018/3059478

**Published:** 2018-10-09

**Authors:** Philippe-Richard Domeyer, Smaragda Ch. Giannakidou, Panagiota Kyriakou, Vasiliki Katsari, Antonios P. Antoniadis, Ioannis K. Lagos, Nikolaos Fragakis, Agoritsa Varaklioti, Vassilios P. Vassilikos

**Affiliations:** ^1^School of Social Sciences, Hellenic Open University, Parodos Aristotelous 18, 26 335 Patra, Greece; ^2^Third Department of Cardiology, Hippokration General Hospital, Aristotle University Medical School, 49 Konstantinoupoleos Road, 54642 Thessaloniki, Greece; ^3^Second Department of Cardiology, Hippokration General Hospital, Aristotle University Medical School, 49 Konstantinoupoleos Road, 54642 Thessaloniki, Greece

## Abstract

**Objective:**

This study aims to (i) translate, culturally adapt, and preliminarily validate the arrhythmia-specific Umea22 (U22) questionnaire and (ii) assess the impact of radiofrequency (RF) ablation and medical treatment on the quality of life of patients with supraventricular tachycardias (SVTs).

**Methods:**

A total of 140 patients with atrioventricular nodal re-entry tachycardia (AVNRT) and atrioventricular re-entry tachycardia (AVRT) were enrolled in the study. Of these, 100 patients underwent RF ablation (group A) and 40 patients were managed with antiarrhythmic medications (group B). Health-related quality of life (HRQoL) was assessed for both groups using the Short Form-36 Health Survey (SF-36) and the arrhythmia-specific Umea22 (U22) questionnaire at baseline and 3-month follow-up. Exploratory and confirmatory factor analyses were performed to assess the validity of the U22 questionnaire. Univariate comparisons of HRQoL scores between study timepoints and multivariate regression analyses adjusting for baseline confounders were conducted.

**Results:**

The factor analysis of the U22 questionnaire yielded a six-factor model (“burden of spells”; “heart contractility”; “character of spells”; “general/non-specific feeling”; “other specific somatic symptoms”; “fear”) with acceptable fit results. Patients of group A showed significant improvement in all SF-36 and U22 scores at 3 months' follow-up compared to baseline (all p<0.05). Patients of group B presented deterioration of the total SF-36 score (p=0.001) and improvement of certain U22 measures, namely, well-being (p=0.004), heartbeat speed, and intensity during arrhythmia spells (p<0.0001 for both measures) at 3 months' follow-up, compared to baseline. Employment status, male sex, and urban residence emerged as important predictors.

**Conclusion:**

The Greek version of the U22 questionnaire is a valid tool to assess SVT-related symptoms. RF ablation appears to exert more pronounced beneficial outcomes on HRQoL of patients with SVTs compared to medical treatment. Prompt referral of patients with SVTs to specialist centers may favorably affect their quality of life and should be encouraged.

## 1. Introduction

Supraventricular tachycardias (SVTs) are cardiac arrhythmias originating from or above the bundle of His. They are typically characterized by narrow (<120 msec) QRS complexes, sudden onset of a rapid heart rate (150-250 beats/min), and duration ranging from a few minutes to a few hours or rarely even a few days. Their clinical manifestation includes palpitations, dizziness, weakness, dyspnea, and less frequently anginal-type chest pain or syncope [1]. Paroxysmal SVTs include atrioventricular nodal re-entry tachycardia (AVNRT) and atrioventricular re-entry tachycardia (AVRT) and are responsible for 60% of all cases [2].

Although SVTs are usually not life-threatening, they are unpredictable in both occurrence and frequency and, apart from cardiac symptoms, can also trigger anxiety and panic attacks [3, 4]. Medical therapy successfully prevents paroxysms of SVTs but commits the patient to long-term medications with potential side-effects. Additionally, up to 50% of patients with SVTs seem to eventually become refractory to medical treatment [5]. Conversely, patients with frequent episodes of AVNRT or AVRT are good candidates for radiofrequency (RF) ablation as a treatment option with more than 90% success rate and low incidence of complications [6]. Therefore, RF ablation provides the opportunity for definitive treatment of SVTs in most affected patients, with excellent success rates and a low risk of procedural complications [7]. For patients, however, who are suboptimal candidates or who refuse to undergo RF ablation, medical therapy can be followed, aiming to suppress recurrent symptomatic episodes of SVTs.

A significant aspect of available treatments for SVTs is their impact on the quality of life of patients. SVTs, like chronic diseases, may lead to psychological decline [8]. The majority of patients experience social isolation and avoid everyday activities, while the rate of absence from work can reach 12% in affected individuals [9]. Therefore, the effects of the available treatment strategies for SVTs on the quality of life of patients warrant further investigation.

This study aimed (i) to translate, culturally adapt, and perform a preliminary validation of the arrhythmia-specific Umea22 (U22) questionnaire and (ii) to evaluate the differential effects of SVT treatment with RF ablation or antiarrhythmic medications on the quality of life of patients and to assess putative predictors of these effects. An important added value of the present study pertains to the concept that differential effects of ablation versus medical therapy in terms of patients' quality of life could lead to an improved decision-making process regarding treatment of supraventricular tachycardias. 

## 2. Methods

### 2.1. Patient Groups and Study Timepoints

We prospectively evaluated the health-related quality of life (HRQoL) in Greek patients with SVTs (either AVNRT or AVRT) treated with RF ablation or medical therapy. A total of 140 consecutive patients aged 16-65 years with newly documented SVTs presenting to two Greek centers from October 2016 to April 2017 were enrolled in the study. All subjects had documented SVTs in either the electrocardiogram (ECG) or Holter monitoring. All subjects were first treated medically and, if the SVT was refractory to medications, they were referred for consideration of RF ablation. Risks and benefits of the procedure were explained to all patients by the treating physicians; patients who consented to undergo an electrophysiological study and RF ablation were enrolled to group A while those who elected to follow medical therapy were enrolled to group B. In group B, the diagnosis was based on clinical features and surface ECG. The first study timepoint for both groups was the initial assessment, i.e., before RF ablation for group A and before initiation of antiarrhythmic medication for group B (baseline). The second study timepoint was at 3 months' follow-up, i.e., 3 months after the RF ablation procedure for group A and 3 months after the initiation of antiarrhythmic medications for group B.

### 2.2. Diagnostic Electrophysiological Study and Radiofrequency Ablation

Patients in group A underwent an electrophysiological (EP) study, which was performed in the fasting state, under local anesthesia. Standard 6Fr bipolar and quadripolar catheters were inserted percutaneously via the right and left femoral vein and positioned in the high right atrium, His bundle region, coronary sinus, and the right ventricular apex. The diagnostic EP study was performed by using a standard protocol [10]. As soon as the clinical tachycardia was induced, detailed mapping and ablation maneuvers were performed as per the standard protocol, using either a conventional or an irrigated-tip catheter. Depending on the underlying mechanism of SVT, successful RF ablation was defined as non-inducibility of AVNRT and loss of delta wave or pathway mediated retrograde ventriculoatrial conduction block for AVRT. In case of a second masked arrhythmia other than the initial one, non-inducibility was required for a successful outcome. A re-evaluation was usually done 30 minutes after ablation during isoproterenol infusion, to confirm procedural success.

### 2.3. Questionnaires

HRQoL was assessed using the Medical Outcomes Study 36-Item Short-Form Health Survey (SF-36) and the arrhythmia-specific Umea22 (U22) questionnaire in both study timepoints. SF-36 includes 35 questions that form 8 subscales, grouped in two component summary scales, namely, mental (vitality, social functioning, emotional role, and mental health) and physical (physical functioning, physical role, bodily pain, and general health). One item, asking about health change, is not included in any subscale. It produces a health profile with scores between 0 and 100 for each dimension [11]. The SF-36 tool has been translated and validated in Greek [12].

The U22 questionnaire was developed for the assessment of symptoms associated with supraventricular tachycardia and includes a total of 22 questions [13]. Questions (Q) concerning general well-being (Q01), effectiveness (Q04) and side-effects (Q05) of pharmacotherapy, the influence of arrhythmia on well-being (Q11), the intensity of discomfort (Q12), and the severity of specific symptoms during a spell (Q13-Q22) are answered on a numerical rating scale with a range of 0-10, with higher scores reflecting a better feeling (Q01) or a more pronounced impact. Question 9 investigates whether arrhythmias initiate suddenly or not. Questions 8 and 10 (quantified as 0-5) estimate the incidence and duration of events, with higher scores denoting higher frequency and duration, respectively. The relevancy of the answers is tested by Q19 about the level of itching, a symptom irrelevant in the context of arrhythmias. Questions 2, 6, and 7 are only answered after a follow-up of more than 3 months and were excluded from the study. Finally, questions 3 to 5 pertain to antiarrhythmic medications and were not included in the analysis.

The translation and cultural adaptation of the U22 questionnaire were done according to existing guidelines [14]. Forward translation using the English version was performed by two independent professional translators. The forward translations were reconciled into a single forward translation by a bilingual expert panel including one of the researchers, in order to resolve any discrepancies and seek agreement. An independent bilingual translator, without knowledge of the original questionnaire, performed the back translation. The expert panel reviewed the modified version. Translation and back-translation did not reveal substantial problems. Next, a cognitive debriefing process was used for the cultural adaptation of the questionnaire as the last step of the translation procedure [15]. As part of this process, the reconciliated Greek version of the U22 was handed over to three arrhythmia experts proficient in English to ensure content validity. Finally, to assess its face validity, the questionnaire was pilot-tested on 26 SVT patients who did not belong to the study groups. During the cultural adaptation process, the questionnaire was found to be overall comprehensible and easy to fill out according to most patients' comments.

The study protocol was approved by the Institutional Ethics Committee. All patients agreed to participate in the study and provided their written informed consent.

### 2.4. Statistical Analysis

#### 2.4.1. Exploratory and Confirmatory Factor Analysis

The U22 questionnaire was psychometrically tested and subscales were created with the use of exploratory factor analysis. Subsequently, confirmatory factor analysis was used to test the fit of the models to our data.

Sample size exceeded the minimum 5 : 1 subjects-to-item ratio necessary for exploratory factor analysis [16]. Furthermore, the Kaiser-Meyer-Olkin Measure of Sampling Adequacy (KMO = 0.689) and Bartlett's Test of Sphericity [*χ*2(120) = 705.428, p < 0.001] indicated that data were adequate for conducting an exploratory factor analysis.

The exploratory factor analysis was carried out using the principal axis factoring method and a subsequent promax rotation. To decide on the number of factors to retain, we used Kaiser's criterion, according to which all factors with eigenvalues<1 are dropped [17]. The rotated factor loadings were computed; the threshold value of 0.4 was adopted for factor extraction [18].

During the confirmatory factor analysis, goodness-of-fit indices which were assessed included (i) the root mean square error of approximation (RMSEA), whose values of 0.06 or less are indicative of an acceptable model fit [19], (ii) the Standardized Root Mean Square Residual (SRMR), whose values of 0.08 or less are indicative of an acceptable model fit [20], and (iii) the Tucker Lewis Index (TLI) and the Comparative Fit Index (CFI), whose values of 0.9 or more are indicative of an acceptable model fit [21, 22].

#### 2.4.2. Assessment of HRQoL Measures

Comparisons of both HRQoL questionnaires scores between study timepoints were performed using the Wilcoxon signed-rank test. Multivariate regression analyses adjusting for baseline confounders were used to examine that differences were independent of any measured confounding effects. Baseline confounders with their respective categories (where applicable) that were included in the multivariate analyses were (i) group (A vs B), (ii) gender (male vs female), (iii) age, (iv) education (<12 years vs >12years), (v) occupation (employed vs any other), and (vi) place of residence (urban vs semi-urban/rural).

The magnitude of change in SF-36 measures was computed using Cohen's d [23], which is a standardized measure of effect size (ES) and provides information on the amount of change in the measure relative to the variation within the measure. Cohen's d is calculated as the difference between the baseline and follow-up scores divided by the standard deviation of baseline scores. According to existing cut-off values for classifying the importance of change, ES may be considered “trivial” (ES<0.20), “small” (ES≥0.20<0.50), “moderate” (ES≥0.50<0.80), or “large” (ES≥0.80) [23]. ES were calculated so that positive values represent improvement and negative values represent deterioration. Since minimal clinically important changes (MCIC) for SF-36 subscales in arrhythmiological settings are not available, we utilized the published standards for minimal “clinically and socially relevant” change in group scores as a measure of MCIC at a group level [24]. These standards at a group level are based on Cohen's d, with minimal important change represented by a moderate effect size (0.50–0.79), which corresponds to at least 5-point change in scores on the 0–100 scale [23].

Statistical analyses were performed using SPSS v.19.0 software (SPSS Inc., Chicago, IL, USA). Two-tailed p values <0.05 were considered statistically significant.

## 3. Results

### 3.1. Exploratory and Confirmatory Factor Analysis

The Cronbach's alpha coefficient for the entire U22 questionnaire was 0.715. Bartlett's test for sphericity was statistically significant (p<0.0001) and the Kaiser-Meyer-Olkin measure of sampling adequacy was 0.689. The factor analysis using promax rotation yielded a six-factor model (Table 1). The first factor denoted by “burden of spells” included items 8, 11, and 12, the second factor entitled “heart contractility” was explained by items 13 and 14, the third factor named “character of spells” consisted of items 9, 10, and 15, the fourth factor labelled “general/non-specific feeling” involved items 1, 18, and 19, the fifth factor identified “other specific somatic symptoms” incorporated items 16, 17, and 20, and the sixth factor designated “fear” was influenced by items 21 and 22.

A confirmatory factor analysis was subsequently run to explore the strength of the proposed 16-item scale, with overall acceptable fit results: RMSEA=0.066, SRMR=0.072, TLI=0.890, and CFI=0.918.

### 3.2. Assessment of HRQoL Measures

Eighty-eight patients in group A had a successful ablation in the first session. The remaining 12 patients required a second ablation, which was performed 1 to 3 months after the first procedure, depending on the timing of symptom reoccurrence.  Table 2 summarizes basic sociodemographic characteristics of the study sample; no statistically significant differences between groups A and B were found at baseline.

At 3 months' follow-up, patients in group A showed a statistically significant increase in the SF-36 total score as well as in all SF-36 scales and subscales (all p<0.0001), whereas patients in group B experienced a statistically significant deterioration in the SF-36 total score, in both physical and mental health status component scales and in most subscales (Table 3). The mean value of the single-item measure of health change showed a statistically significant increase in group A (3.78±0.75 vs 1.75±0.91, p<0.0001), whereas no significant change was noted in group B (3.95±0.88 vs 3.70±1.04, p=0.165). The results of the multivariate analyses, adjusting for baseline confounders, are shown in Table 4.

With regard to the U22 questionnaire, all measures improved statistically significantly at the 3-month follow-up compared to baseline for group A, whereas in group B such an improvement was only documented in the general well-being and the heartbeat's speed and intensity during arrhythmia spells (Table 5). The results of the multivariate analyses, adjusting for baseline confounders, are shown in Table 6.

## 4. Discussion

SVTs are common arrhythmias affecting a considerable number of patients in Greece, with a prevalence of up to 3.000 individuals per year [25]. Despite their mostly benign nature, they result in a significant impairment of health-related quality of life [9, 26–28] and are associated with an increased economic burden [29].

This is the first study aiming to assess the impact of RF ablation and pharmacotherapy on HRQoL indices in a Greek population sample. For this purpose, the concomitant use of generic and disease-specific instruments seemed most appropriate. Since no arrhythmia specific tool existed in the Greek language, the U22 questionnaire, developed to quantify symptoms associated with supraventricular tachycardia [30], was chosen, translated, and culturally adapted to the Greek population, showing good face and content validity. The results of the subsequent exploratory factor analysis revealed a 6-factor model, whose fit to the data was found to be acceptable, according to the confirmatory factor analysis. This is the first study to perform such a validation process on the U22 questionnaire; as a result, comparisons of the results with other studies were not feasible.

The study results denoted that RF ablation and antiarrhythmic medications have a totally different impact on the quality of life of patients with SVTs. Importantly, in the RF ablation group, all SF-36 indices exhibited both statistically highly significant and clinically meaningful improvements at the 3-month follow-up measurement compared to baseline. These improvements were further confirmed by the outcomes of U22 measurements, according to which patients declared fewer symptoms, decreased frequency, and limited duration of episodes at the second measurement. On the other hand, in the pharmacotherapy group, most of the SF-36 indices denoted statistically significant deteriorations, though this was clinically evident only in the General Health subscale, according to Cohen's d statistic. However, even a small effect size of 0.2, which is a cut-off that was surpassed by seven out of eleven SF-36 measures in group B, may not be too small as to be trivial [31]. Importantly, the intergroup differences were confirmed by the multivariate analyses. It is worth mentioning that previously published studies have also documented a statistical superiority of RF ablation over pharmacotherapy in improving quality of life indices [9, 13, 26, 32–35]; however none of those reported any measures assessing the existence of clinically significant differences. In addition, to our knowledge, this is the first study showing that patients in the medical control group may even exhibit a deterioration in SF-36 indices in such a short time period. Nevertheless, this interesting observation was not confirmed by U22 measurements; therefore, it should be interpreted with caution. A similar study from Lau et al [33] showed no change in the quality of life of the medical control group; however the extremely small number of participants in the control group (9 patients) may have considerably limited the study's power.

Making one step beyond the demonstration of significant changes, multivariate analyses were performed to investigate the presence of predictors able to modify the difference in responses of patients in terms of HRQoL at baseline and follow-up, in both groups. Interestingly, patients currently employed declared a lesser improvement in the SF-36 Role Physical, SF-36 General Health, and U22 “arrhythmia events affect well being” measures, compared to others. This interesting correlation might reflect a partial “masking” of the beneficial effect of the intervention in the case of employed patients, due to their increased physical or mental workload and associated anxiety, compared to others. In addition, male gender was significantly associated with a less pronounced improvement in the physical functioning scale but a sharper decrease of the “fatigue” during a spell (Q18), compared to women. Although overall physical functioning assessed by a general scale is not necessarily correlated to fatigue experienced during a spell as estimated by a disease-specific scale, other studies are needed to further investigate and replicate this intriguing finding. The results of existing studies are discordant; Farkowski* et al.* [36] reported a higher severity of symptoms in females compare to males, both at baseline and 2 months after RF ablation, whereas in the study of Meissner et al [35] gender was found to not have a significant influence on HRQoL measurements. However, none of the existing studies used a multivariate approach, modelling differences in HRQoL indices. Finally, urban residence was associated with a more pronounced decrease in the “fear to others” measure, compared to semi-urban or rural residence. This peculiar finding has not been reported in the literature before and warrants further investigation, although the possible impact of unmeasured confounders cannot be excluded.

This study has certain limitations that should be addressed. First, it was conducted in the largest RF center of the study region, with considerable expertise in SVTs RF ablation [37]; however, the generalizability of the findings to the entire region cannot be documented, as patients from other centers were not recruited. Also, the short duration of the study does not enable accurate conclusions for the differential long-term impact of RF ablation and antiarrhythmic medications on the quality of life of patients. Moreover, only a preliminary validation of the U22 questionnaire was performed, which did not include all of the U22 items, as per the study's design. Furthermore, as the RF ablation's failure rate was low, we were not able to estimate whether RF ablation failure could have a negative impact on HRQoL scores. Additional sociodemographic and clinical characteristics of the study population, e.g., marital status, income, access to primary care, categorization to AVNRT and AVRT groups, arrhythmia episode frequencies, and antiarrhythmic medications used in group B and comorbidities, could have affected the study results. Unfortunately, such information was not available and therefore not included in this study.

Another limitation of the study pertains to its non-randomized design. The two study groups did not differ in terms of examined confounders, namely, gender, age, profession, and place of residence, and any differences in terms of baseline quality of life were accommodated in the analysis, as the latter was based on the multivariate analysis of differences and not on absolute values. Notably, the documented changes persisted after adjustment for the examined confounders, with the exception of items 13 (heartbeat speed) and 14 (heartbeat intensity) of the U22 questionnaire, where no statistically significant predictors emerged. However, the effects of differences in unmeasured confounders cannot be ruled out.

To conclude, this study not only confirms the benefits from RF ablation in terms of quality of life improvement and symptom relief, but also suggests that substituting RF ablation by medical treatment may adversely and rapidly affect the patients' HRQoL. We believe that this study highlights the need for a more robust recommendation for early referral of patients from primary care physicians to specialist centers.

## 5. Conclusions

SVTs are common arrhythmias, considerably affecting the patient's quality of life. The Greek version of the U22 questionnaire is a psychometrically sound tool to assess SVT-related symptoms. RF ablation is a first-line therapy for the vast majority of patients with SVTs, which appears to have more pronounced benefits in HRQol than medical therapy at 3-month follow-up regarding Greek patients with SVTs. Large scale, prospective randomized studies are warranted to further investigate the long-term effects of RF ablation compared to medical treatment on the quality of life of patients with SVTs as well as to assess the cost-effectiveness of both approaches.

## Figures and Tables

**Table 1:** Factor analysis with polychoric correlations (rotated factor loadings^a^).

**Measure**	**Item**	**Factor 1**	**Factor 2**	**Factor 3**	**Factor 4**	**Factor 5**	**Factor 6**
Well-being	1	-0.436	0.113	0.224	**-0.739**	0.082	-0.019
Arrhythmia frequency	8	**0.622**	-0.105	0.232	0.192	0.001	-0.446
Arrhythmia sudden onset	9	0.372	-0.007	**0.551**	-0.328	0.056	0.052
Arrhythmia duration	10	0.374	-0.046	**0.469**	-0.084	-0.192	0.380
Arrhythmia events affects well-being	11	**0.822**	0.049	-0.020	0.135	0.015	0.131
Discomfort	12	**0.877**	0.090	-0.024	0.028	0.041	0.086
Heartbeat speed	13	0.066	**0.960**	-0.032	-0.055	0.017	-0.012
Heartbeat intensity	14	0.023	**0.952**	0.001	-0.026	0.033	0.031
Heartbeat irregularity	15	-0.023	-0.020	**0.776**	0.104	0.043	-0.036
Faint or dizziness	16	0.118	0.137	-0.030	0.164	**0.660**	-0.067
Pain	17	0.046	-0.022	-0.056	-0.205	**0.733**	0.369
Fatigue	18	-0.311	0.125	0.376	**0.547**	-0.250	0.147
Pruritus	19	0.169	0.205	0.249	**0.474**	0.092	0.005
Dyspnea	20	-0.178	-0.043	0.440	-0.048	**0.529**	-0.228
Personal fear	21	0.186	0.030	-0.015	0.255	0.086	**0.725**
Fear to others	22	-0.136	-0.161	0.110	0.358	0.263	**0.447**

^a^Numbers in bold indicate the highest factor loadings for each item.

**Table 2:** Sociodemographic characteristics of patients with SVT.

	Group A^∗^	Group B^∗^	P^∗∗^
Number	100	40	
Gender			
*Males*	43 (43.0%)	19 (47.5%)	0.629
*Females*	57 (57.0%)	21 (52.5%)	0.629
Age (yrs)	46.55±11.4	43.55±11.18	0.160
Education			
*Primary school*	14 (14.0%)	4 (10.0%)	0.525
*Secondary school*	14 (14.0%)	7 (17.5%)	0.602
*High school*	40 (40.0%)	16 (40.0%)	1.000
*Bachelor's*	28 (28.0%)	12 (30.0%)	0.814
*Master's*	1 (1.0%)	0 (0.0%)	n/a
*Ph.D.*	2 (2.0%)	1 (2.5%)	0.682
*Unspecified*	1 (1.0%)	0 (0.0%)	n/a
Occupation			
*Employee*	45 (45.0%)	21 (52.5%)	0.424
*Self-employed*	23 (23.0%)	10 (25.0%)	0.802
*Unemployed/retired*	9 (9.0%)	5 (12.5%)	0.534
*Student*	5 (5.0%)	1 (2.5%)	0.511
*Household*	18 (18.0%)	4 (10.0%)	0.242
Place of residence			
*Urban*	44 (44.0%)	16 (40.0%)	0.669
*Semi-urban/rural*	56 (56.0%)	24 (60.0%)	0.669

n/a: not available.

^∗^Data expressed as frequencies (percentages) for categorical variables and mean ± SD for continuous variables.

^∗∗^Chi-squared test and t-test for categorical and continuous variables, respectively.

**Table 3:** SF-36 measures at baseline and follow-up.

Measure	Group	Baseline mean±SD	Follow-up mean±SD	Change mean±SD	ES	P^∗^
PF	A	66.70±26.07	87.05±20.28	20.35±21.94	0.78	**<0.0001**
	B	57.25±24.31	59.25±21.35	2.00±20.81	0.08	**<0.0001**
RP	A	40.75±32.69	78.50±32.38	37.75±37.01	1.15	**<0.0001**
	B	36.25±29.39	29.38±25.87	-6.87±35.35	-0.23	0.249
BP	A	76.15±23.53	92.13±17.11	15.98±18.94	0.68	**<0.0001**
	B	75.38±23.01	68.16±18.58	-7.22±22.75	-0.30	**0.007**
GH	A	69.09±18.30	79.96±18.55	10.87±13.95	0.59	**<0.0001**
	B	61.38±17.89	49.83±23.25	-11.55±22.27	-0.65	**0.005**
VT	A	48.90±7.96	59.05±9.66	10.15±8.72	1.28	**<0.0001**
	B	47.50±6.79	47.13±7.24	-0.37±12.06	-0.05	**0.002**
SF	A	57.38±26.66	82.25±22.56	24.87±27.27	0.93	**<0.0001**
	B	48.44±26.88	43.44±25.63	-5.00±22.07	-0.19	**<0.0001**
RE	A	41.00±33.79	77.00±32.38	36.00±44.36	1.07	**<0.0001**
	B	40.00±34.76	35.83±28.63	-4.17±38.63	-0.12	0.093
MH	A	50.80±9.95	58.44±11.45	7.64±9.52	0.77	**<0.0001**
	B	50.00±9.53	47.60±8.39	-2.40±9.01	-0.25	**0.001**
PCS	A	67.41±18.06	84.73±16.46	17.32±13.84	0.96	**<0.0001**
	B	60.58±18.27	55.20±17.26	-5.38±17.19	-0.29	**<0.0001**
MCS	A	50.54±8.12	63.05±9.97	12.51±9.27	1.54	**<0.0001**
	B	48.57±7.88	46.21±9.02	-2.36±9.67	-0.30	**0.026**
Total score	A	61.54±12.35	75.29±11.08	13.75±9.70	1.11	**<0.0001**
	B	54.47±12.19	50.62±12.24	-3.85±12.16	-0.32	**0.001**

^*∗*^Wilcoxon signed rank test.

ES=effect size, PF = physical functioning, RP = role physical, BP = bodily pain, GH = general health, VT = vitality, SF = social functioning, RE = role emotional, MH = mental health, PCS= physical component scale, MCS= mental component scale.

**Table 4:** Multivariate linear regression analyses of the change in SF-36 measures at follow-up vs. baseline^∗^.

Measures	Mean change±SD	Category	Coeff. (95% CI)	P
PF	15.11±23.10			
*Group*		*A vs B*	13.13 (11.24, 27.02)	<0.0001
*Sex *		*Male vs Female*	-12.12 (-19.91, -4.34)	0.003
RP	25.00±41.66			
*Group*		*A vs B*	43.74 (30.21, 57.28)	<0.0001
*Occupation*		*Employed vs other*	-17.31 (-32.09, -2.53)	0.022
GH	4.46±19.52			
*Group*		*A vs B*	21.42 (15.21, 27.63)	<0.0001
*Occupation*		*Employed vs other*	-8.84 (-15.63, -2.06)	0.011

PF = physical functioning, RP = role physical, GH = general health.

^∗^In multivariate linear regression analyses of all other measures (not shown), *Group* was the only statistically significant predictor (p<0.0001 in all analyses).

**Table 5:** Umea22 measures at baseline and follow-up.

Measure	Item	Group	Baseline mean±SD	Follow-up mean±SD	Change mean±SD	P^∗^
Well-being	01	A	2.17±2.08	6.82±2.23	4.65±3.24	**<0.0001**
		B	2.18±1.65	3.28±2.00	1.10±2.25	**0.004**
Arrhythmia frequency	08	A	2.48±1.11	0.94±0.94	-1.54±1.76	**<0.0001**
		B	2.38±1.27	2.18±1.03	-0.20±1.34	0.437
Arrhythmia duration	10	A	2.47±1.21	0.70±1.05	-1.77±1.54	**<0.0001**
		B	2.30±1.29	2.25±1.17	-0.05±1.01	0.706
Arrhythmia events affect well-being	11	A	8.00±2.15	4.14±2.67	-3.86±2.66	**<0.0001**
		B	7.73±2.21	8.05±1.77	0.32±2.068	0.639
Discomfort	12	A	8.18±2.04	4.21±2.84	-3.97±2.87	**<0.0001**
		B	8.03±2.14	8.18±1.50	0.15±2.18	0.848
Heartbeat speed	13	A	9.22±1.34	7.45±2.40	-1.77±2.264	**<0.0001**
		B	9.35±0.89	8.00±1.20	-1.35±1.17	**<0.0001**
Heartbeat intensity	14	A	9.10±1.47	7.24±2.51	-1.86±2.35	**<0.0001**
		B	9.23±0.89	8.08±1.12	-1.15±1.37	**<0.0001**
Hearbeat irregularity	15	A	6.56±3.69	4.23±3.98	-2.33±3.48	**<0.0001**
		B	5.73±3.73	6.08±3.10	0.35±3.59	0.907
Faint or dizziness	16	A	4.86±3.67	1.89±2.58	-2.97±3.67	**<0.0001**
		B	4.85±3.36	4.93±2.72	0.08±3.025	0.744
Pain	17	A	3.83±3.40	1.43±2.41	-2.40±3.50	**0.041**
		B	3.10±3.25	3.15±2.61	0.05±2.21	0.901
Fatigue	18	A	1.30±3.03	0.76±2.13	-0.54±2.87	**<0.0001**
		B	1.18±2.82	1.75±3.45	0.57±2.61	0.236
Pruritus	19	A	7.55±2.88	3.77±2.81	3.78±3.59	**<0.0001**
		B	7.70±2.45	7.20±2.04	-0.50±2.55	0.098
Dyspnea	20	A	4.33±3.80	1.69±2.42	-2.64±3.17	**<0.0001**
		B	3.78±3.28	3.43±2.80	-0.35±2.29	0.166
Personal fear	21	A	6.85±2.70	3.61±2.60	-3.23±2.62	**<0.0001**
		B	6.75±2.80	6.93±1.93	0.18±2.70	0.796
Fear to others	22	A	6.03±3.06	3.93±3.08	-2.08±2.83	**<0.0001**
		B	7.05±2.52	7.35±2.37	0.30±2.57	0.419

^*∗*^Wilcoxon signed rank test.

**Table 6:** Multivariate linear regression analyses of the change in Umea22 measures at follow-up vs. baseline^∗^.

Measures	Mean change±SD	Category	Coeff. (95% CI)	P
Arrhythmia events affect well being	-2.64±3.16			
*Group*		*A vs B*	-4.10 (-5.03, -3.16)	<0.0001
*Occupation*		*Employed vs other*	1.18 (0.15, 2.21)	0.025
Fatigue	-0.23±2.83			
*Group*		*A vs B*	-1.19 (-2.21, -0.16)	0.024
*Sex *		*Male vs Female*	-1.73 (-2.75, -0.72)	0.001
Fear to others	-1.40±2.96			
*Group*		*A vs B*	-2.41 (-3.43, -1.40)	<0.0001
*Residence*		*Urban vs other*	-1.31 (-2.26, -0.36)	0.008

^*∗*^In multivariate linear regression analyses of all other measures (not shown), *Group* was the only statistically significant predictor (p<0.0001 in all analyses), except for items 13 (heartbeat speed) and 14 (heartbeat intensity), where no statistically significant predictor emerged.
